# Towards Predicting the Response of a Solid Tumour to Chemotherapy and Radiotherapy Treatments: Clinical Insights from a Computational Model

**DOI:** 10.1371/journal.pcbi.1003120

**Published:** 2013-07-11

**Authors:** Gibin G. Powathil, Douglas J. A. Adamson, Mark A. J. Chaplain

**Affiliations:** 1 Division of Mathematics, University of Dundee, Dundee, United Kingdom; 2 Department of Clinical Oncology, Ninewells Hospital and Medical School, Dundee, United Kingdom; Vanderbilt University Medical Center, United States of America

## Abstract

In this paper we use a hybrid multiscale mathematical model that incorporates both individual cell behaviour through the cell-cycle and the effects of the changing microenvironment through oxygen dynamics to study the multiple effects of radiation therapy. The oxygenation status of the cells is considered as one of the important prognostic markers for determining radiation therapy, as hypoxic cells are less radiosensitive. Another factor that critically affects radiation sensitivity is cell-cycle regulation. The effects of radiation therapy are included in the model using a modified linear quadratic model for the radiation damage, incorporating the effects of hypoxia and cell-cycle in determining the cell-cycle phase-specific radiosensitivity. Furthermore, after irradiation, an individual cell's cell-cycle dynamics are intrinsically modified through the activation of pathways responsible for repair mechanisms, often resulting in a delay/arrest in the cell-cycle. The model is then used to study various combinations of multiple doses of cell-cycle dependent chemotherapies and radiation therapy, as radiation may work better by the partial synchronisation of cells in the most radiosensitive phase of the cell-cycle. Moreover, using this multi-scale model, we investigate the optimum sequencing and scheduling of these multi-modality treatments, and the impact of internal and external heterogeneity on the spatio-temporal patterning of the distribution of tumour cells and their response to different treatment schedules.

## Author Summary

Anti-cancer treatments such as radiotherapy and chemotherapy have evolved through clinical trial-and-error over decades, and although they cure some cases and are partially effective in many, the majority of such cancers ultimately recur. Doctors turn to new, expensive drugs as they emerge, but perhaps fail to study and learn how to use the therapies they already have most effectively. This is partly because clinical trials are expensive to conduct, both in terms of time and money. The cancer cell is complicated, but many mechanisms that control its response to treatment are now understood. We show here how a mathematical model accurately reproduces the results of previous biological experiments of cancer treatment, opening up the possibility of using it to predict which combinations of drugs and radiotherapy would be best for patients.

## Introduction

Chemotherapy and radiotherapy play important roles in the primary treatment of many cancers and in improving the survival after cancer surgery. Currently, numerous chemotherapeutic drugs and irradiation techniques are employed, which have evolved over several decades through empirical clinical usage. New treatments, such as a novel drug or a change in the scheduling of radiotherapy take many years to assess by conducting a clinical trial and clinicians would benefit greatly from having an alternative scientific approach to decide on how to improve current treatment strategies, but also in arriving at good decisions more quickly. Mathematical modelling of such complex, dynamic situations might provide one solution to this problem, and speed up delivery of efficacious treatments to patients while preventing the use of potentially sub-optimal treatment combinations. The effectiveness of these treatment protocols is considerably affected by internal tumour heterogeneities caused by perturbations in the intracellular pathways as well as by dynamical changes in the tissue microenvironment, in particular the distribution of oxygen [1]. Hence, it is important to consider such heterogeneity when studying various optimisation protocols, as this can help in improving the delivery of multi-modality treatments.

A common treatment modality for cancer is chemotherapy. Its delivery is limited by toxicity to normal tissues, so is often delivered in cycles that allow recovery of normal cells, but also, unfortunately, of tumour cells, leading to treatment failure. Chemotherapeutic drugs function by killing the tumour cells through interfering with the cell-cycle mechanism, which regulates complex intracellular processes such as proliferation, cell division and DNA replication [2]. The cell-cycle mechanism is very dynamic in nature and is influenced by the surrounding microenvironment, which contributes to cell-cycle mediated drug resistance and poor treatment outcome [1], [2]. One way of overcoming this is by using an appropriate combination of chemotherapeutic drugs that target the cell at various cell-cycle phase points, thus interfering with tumour cell division. Radiotherapy is curative for certain cancers when used as the sole treatment, but clinical trials conducted in the last thirty years suggest a synergistic effect of concomitant chemotherapy and radiotherapy. As with chemotherapy, the cell-cycle plays a vital role in mediating a cell's sensitivity towards radiation therapy, as the cell-cycle phase determines the cell's relative radiosensitivity [3], [4]. Moreover, various studies have shown that the cells that are in G2-M phase are more sensitive to the radiation than those that are in G1 phase [3]. Furthermore, irradiation can also alter a cell's cell-cycle dynamics through the activation of various intracellular pathways including the p53 and p21 pathways [4]. Activation of these pathways and related cell repair mechanisms can delay the rate of progression of a cell's cell-cycle, causing a group of tumour cells to accumulate either in the G1 or G2 phase, preventing them from undergoing mitosis and making them progress in a synchronous manner [3], [4].

The treatment-dependent perturbations of cell-cycle progression together with cell-cycle-dependent therapeutic sensitivity are some of the many rationales behind the use of kinetically-based administration protocols of chemotherapy and radiation therapy [3], [5], [6]. Studies have shown that both radiation therapy and chemotherapeutic drugs can induce a cell-cycle synchrony and arrest cells at a particular cell-cycle phase which improves the effectiveness of the next dose of radiation/chemotherapy [3], [5]. For example, while the drug paclitaxel induces a cell-cycle arrest at the cell-cycle phase G2-M, Flavopiridol causes cells to accumulate in G1 and G2 phases, enhancing the radiation sensitivity [3]. Alternatively, radiation-induced cell-cycle delay can help various cell-cycle phase-specific drugs to induce a higher cell kill. Moreover, combination regimes can also provide benefit from spatial cooperation and tissue reoxygenation, which enhance therapeutic response. As the interdependency of such therapeutic protocols, the cell-cycle mechanism and the tumour microenvironment clearly affect a cell's response to therapy, it is important to carefully study optimal combination and sequencing of treatments in order to help clinicians design therapeutic protocols that improve survival rates, in which mathematical modelling can be very helpful.

Clinically driven mathematical models can be used as powerful tools to understand, study, and provide useful predictions related to the outcome of various treatment protocols used to treat human malignancies. Although there are several models in the literature that study chemotherapy and radiation therapy, very few of them analyse the effect of the cell-cycle in treatment response [7]–[14]. Recently, Powathil et al. [15] developed a hybrid multiscale cellular automaton model incorporating the effects of oxygen heterogeneity and cell-cycle dynamics to study cell-cycle based chemotherapy delivery. They have shown that an appropriate combination of cell-cycle specific chemotherapeutic drugs could effectively be used to control tumour progression. Most of the mathematical models for radiation therapy are based on a linear quadratic (LQ) formulation [12], [14], [16]–[20]. A brief summary of various approaches in modelling tumour dynamics and radiotherapy can be found in the review by Enderling et al. [21]. Here, we use a discrete multiscale modelling approach to study the multiple effects of cell-cycle and radiotherapy. Ribba et al. [11] proposed a multiscale model incorporating a discrete mathematical model for cell-cycle regulation and cell-cycle phase dependent radiation sensitivity. Richard et al. [12] studied *in vitro* responses of cells with cell-cycle phase-specific sensitivity to targeted irradiation and analysed the so called “bystander effect” using a cellular automaton approach.

In the present paper, we study the multiple effects of radiation therapy when applied in combination with cell-cycle specific chemotherapy in the control of malignant cell growth by using a previously developed hybrid multiscale cellular automaton model for tumour cell growth [15]. In particular, we are interested in studying the effects of cell-cycle regulation in radiation therapy and further, how radiation-induced cell-cycle heterogeneity can potentially be used to increase tumour control when radiotherapy is administered with chemotherapy (“chemoradiotherapy”). Moreover, as the radiation sensitivity is also affected by the surrounding tumour microenvironment, especially the oxygen distribution, we use a modified linear quadratic model to study the effects of radiation in a changing microenvironment.

## Results/Discussion

For many years clinicians have been using chemo-radiotherapeutic combinations for treating various human cancers, as these interact with each other to provide better treatment outcomes [3], [5], [6]. One of the significant factors that can affect the interaction between these modalities and their effectiveness is the intracellular cell-cycle dynamics [1]–4. In an earlier study [15], we analysed the effects of cell-cycle phase-specific chemotherapeutic drugs in controlling tumour growth using a hybrid multiscale model. Here, we have incorporated a detailed model for radiation damage within the hybrid multiscale framework to study the cell-cycle dynamics and their effects during the combined treatment protocols. In the following description, we discuss the computational results that we obtained from the mathematical model that incorporated internal cell-cycle dynamics and oxygen heterogeneity to study multiple therapeutic responses. The details of the hybrid multiscale mathematical model, incorporating the effects of radiation and chemotherapies are given in the methods section.

### Effects of cell-cycle based chemotherapy

Cell-cycle phase-specific chemotherapeutic drugs are used in treating various human malignancies as they interfere with the rapidly proliferating mass of the cells by blocking their cell division cycle. Some of these chemotherapeutic drugs are S phase-specific as they interfere with its replication (e.g. topoisomerase or thymidylate synthase inhibitors), resulting in cell death or cell-cycle arrest at the intra-S checkpoint or at the G2/M checkpoint. Some other drugs are M phase-specific as they damage the formation of the mitotic spindle or prevent it from disassociating (e.g. taxanes, vinca alkaloids) while some block phase transitions at G1/S or G2/M cell-cycle checkpoints (e.g. CDKIs) and other drugs are not necessarily phase-specific as they interact with the DNA irrespective of its cell-cycle phases. Here, for simplicity we consider two types of phase-specific chemotherapeutic drugs that are either G1 specific or G2-S-M specific. While the concept of phase-specific chemotherapy is useful, and although some drugs have specific effects on the machinery of mitosis (e.g. ‘spindle’ poisons) it is becoming clear that chemotherapy drugs may affect more than one aspect of the cell cycle, and so the concept of phase-specificity is somewhat of an over-simplification.

The effects of cell-cycle specific chemotherapeutic drugs on solid tumours with intracellular and oxygen heterogeneities are described using the same cellular automaton framework used by Powathil et al. [15]. Using the mathematical model we have shown that the cytotoxic effectiveness of the cell-cycle phase-specific chemotherapeutic drugs is significantly dependent on the spatial distribution of the tumour cell mass, the timing of the drug delivery, the time between the doses of cytotoxic drugs, and also the cell-cycle and oxygen heterogeneity [15]. We have assumed that the diffusion coefficients and supply rates depend on the location of tumour cells within the tumour, as observed experimentally and hence, the cell-kill due to the chemotherapeutic drugs that are introduced affect the diffusion and supply rates of the drug and nutrients in a favourable manner and thus help to redistribute the subsequent doses introduced [15]. The study also highlighted the importance of considering intracellular and external heterogeneities while studying the potential effectiveness of chemotherapeutic drugs [15].

### Effects of radiation therapy: Cell-cycle, hypoxia and radiation sensitivity

The effectiveness of radiation therapy significantly depends on the intracellular and extracellular dynamics of the targeted tumour. The key intracellular processes, such as cell-cycle dynamics and external factors including oxygen distribution play a vital role in determining the radiosensitivity of the cells that are irradiated [3], [22]. In addition, the radiation fractions (treatments) that are delivered further dynamically change this radiosensitivity over time by redistributing the tumour cells within the cell-cycle, by inducing repopulation of the tumour cell mass, by allowing reoxygenation of the tumour, and by causing the need for repair of the DNA damage induced by treatment [3], [4], [22].

#### Cell-cycle phase redistribution

**Figure 1:**
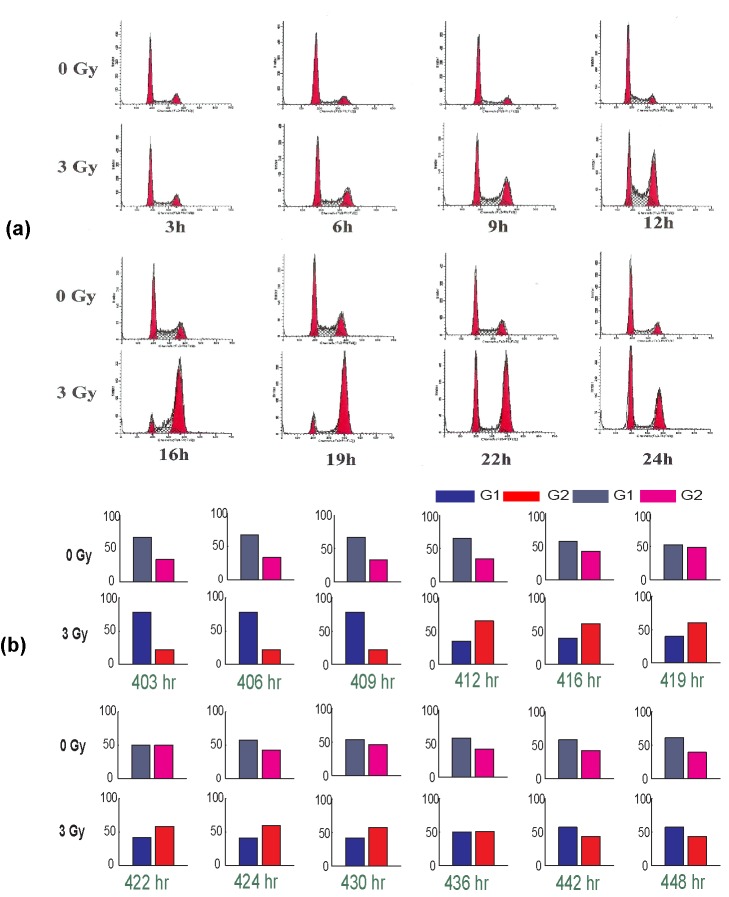
Comparison of the cell-cycle distributions with and without radiation. (a) experimental results from Chaudhry [23] and (b) simulation results. (a) Cell cycle distribution of HeLa Cells after radiation exposure. Cells were irradiated with 3 Gy and samples were taken after 3 h, 6 h, 9 h, 12 h, 16 h, 22 h, and 24 h for flow cytometry analysis. The cell cycle distribution of irradiated cells was compared to unirradiated cells collected at the same time points. The left peak in each case represents cells in G1 and the right peak represents cells in G2 phase. From Chaudhry [23] (BioMed Central OpenAccess). (b) The total number of cells in G1 and G2 phases when the cell is irradiated with dose = 3 Gy at time = 400, 403, 406, 409, 412, 416, 419, 422, 424, 430, 436, 442 and 448 h. The cell-cycle distribution of irradiated cells was compared to unirradiated cells collected at the same time points. The results show a qualitative agreement with the experimental results.

Experimentally, it has been shown that ionizing radiation can slow down the rate of growth of the cell populations, blocking them in various phases of the cell-cycle, resulting in a cell-cycle phase redistribution leading to a partial synchronisation [3], [23], [24]. Figure 1a (and Figure S1) shows the experimental results from a base excision repair study by Chaudhry [23] (Figure 5 in [23]) which shows the cell-cycle distribution of HeLa cells after radiation exposure. In this experimental study, HeLa cells were irradiated with 3 Gy (1 Gray = 1 J/kg) of radiation and the cell-cycle distribution of cells sampled at 3 h, 6 h, 9 h, 12 h, 16 h, 19 h, 20 h, 22 h and 24 h were analysed using flow cytometry [23]. The results obtained after irradiation were also compared against a control population (no radiation) sampled at the same time points. The results show that the controls have cells predominantly distributed in G1 as compared to G2. After irradiation, the majority of the cells start to accumulate in G2 phase, about 12 h after irradiation and stay in G2 phase before going back to a G1 phase dominant cell distribution by 22–24 h after irradiation. In another experimental study by Goto et al. [24], where human TK6 lymphoblastoids are irradiated with a 3 Gy X-ray radiation, a similar change in cell-cycle distribution was also reported (Figure 5 in [24]). The results from Goto et al. [24] show that at about 6 h after the irradiation, the majority of the cells that were in G1 accumulate in G2 and stay there for up to 36 h until returning to the G1 phase of the cell-cycle [24].

**Figure 5:**
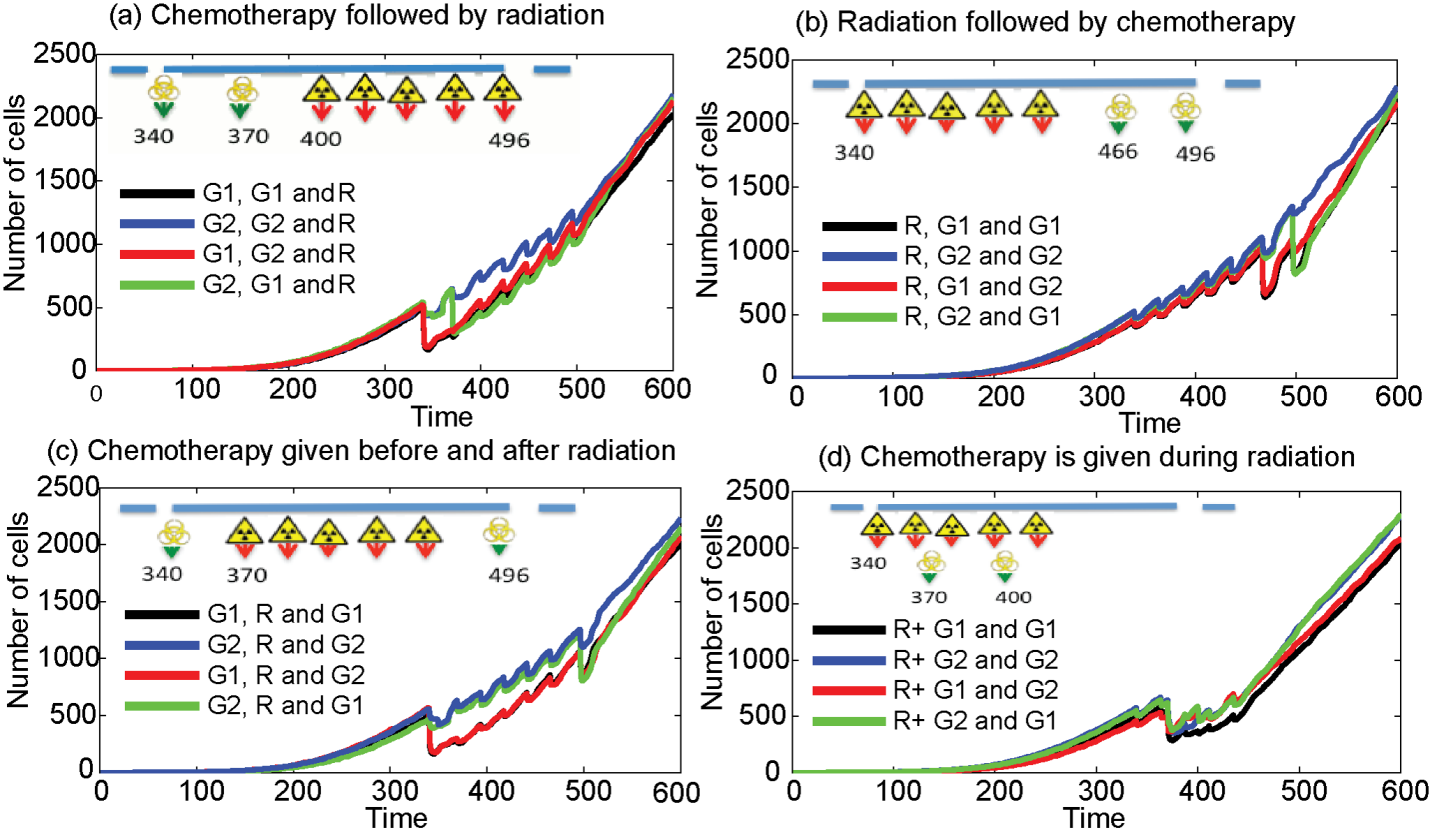
Number of cells with different combinations of chemotherapy and radiation therapy. Two doses of G1 and/or G2 drugs are given in a combination with radiation therapy given in 5 fractions (1 week) with a daily dose of 2.5 Gy. (a) Plots when two doses of drugs are given (time = 340 h, 370 h) before radiation (time = 400 h), (b) plots when two doses of drugs are given (time = 466 h, 496 h) after radiation (time = 340 h), (c) plots when each doses of drugs are given before (time = 340 h) and after (time = 496 h) radiation (time = 370 h) and (d) plots when two doses of drugs are given (time = 370 h and 400 h) during radiation (time = 340 h).

We have analysed these changes in cell-cycle phase distribution caused by radiation using our multiscale mathematical model and compared the results against the control population (without radiation). To compare with the experimental data, the tumour cells are allowed to grow (starting with one tumour cell at time zero) until time = 400 h (about 1000 cells) and then the cells are irradiated at time = 400 h with a radiation dose of 3 Gy and the the corresponding number of cells in G1 and G2 phases are plotted in Figure 1b at times 403 h, 406 h, 409 h, 412 h, 419 h, 422 h, 424 h, 430 h, 436 h, 442 h and 448 h. The plots in Figure 1b show that after irradiation at 400 h, the majority of the cells stay in G1 phase up to 409 h. By 412 h after the radiation, most of the cells enter G2 phase of the cell-cycle, making the number of cells in G2 phase higher than the number in G1 phase. The cells maintain this state until 436 h and then they return back to a G1 phase dominant cell distribution. However, in the control population, the majority of the cells are distributed in the G1 phase of the cell-cycle throughout the sampling times.

These results show a qualitative agreement with the experimental studies described above [23], [24] and indicate that the developed model can satisfactorily predict qualitative changes in the cell-cycle distribution during radiation therapy. This redistribution of cells within their cell-cycle phases can significantly affect response to subsequent doses of radiation therapy or chemotherapy, but at the same time any additional doses of radiation can cause further redistribution of cells in the cell-cycle. In the case of irradiation of cells with one single dose, a subsequent dose of G2 phase-specific chemotherapy may seem a better choice if it is given within the time frame of 12–36 h after the radiation as the number of cells in G2 phase is higher than the number in G1 phase. However, this benefit may not be seen if multiple fractions of radiation doses are given, as the cell-cycle dynamics change accordingly.

#### Cell-cycle and radiation dosage

**Figure 2:**
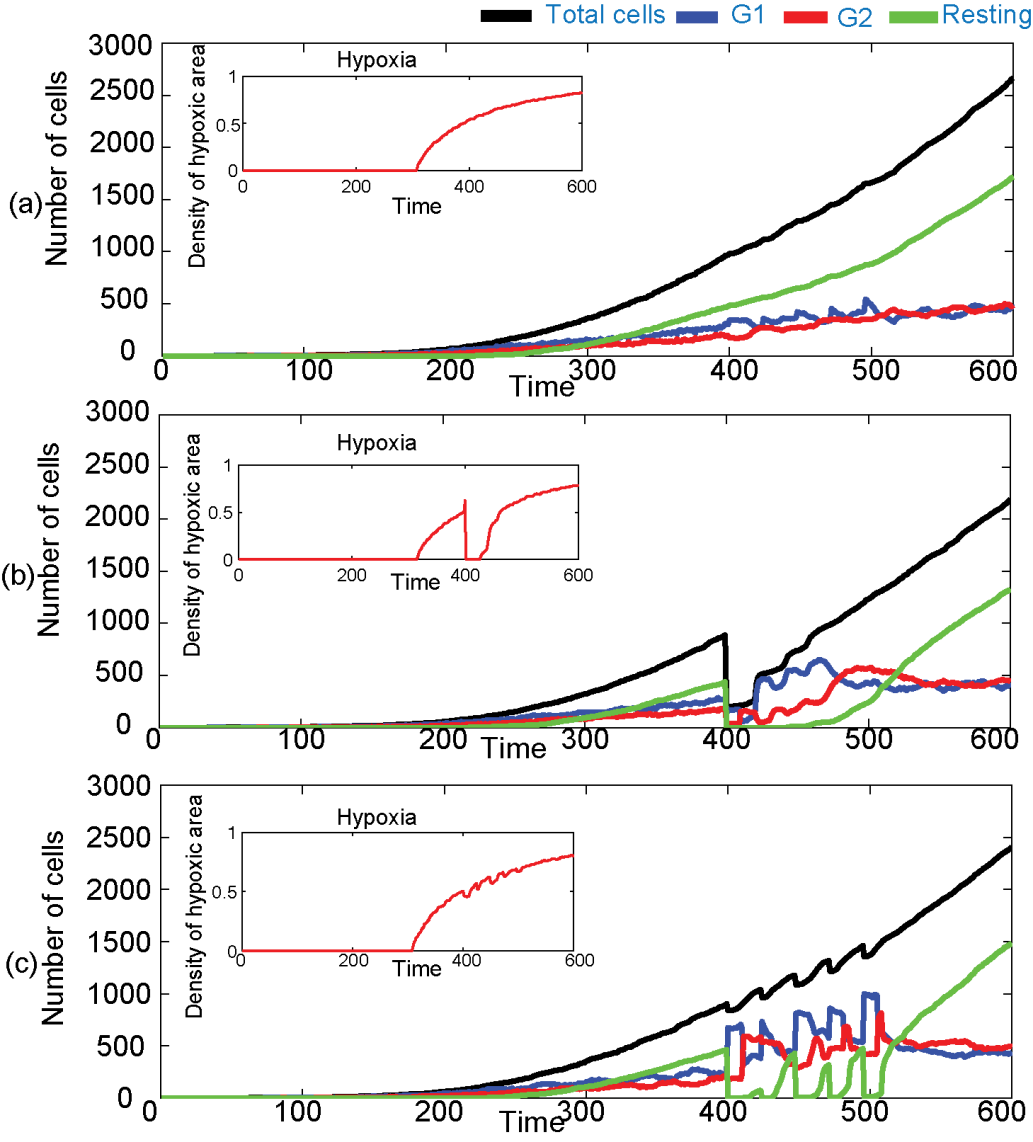
Number of cells in G1, G2 and resting phases for a heterogeneous environment. The number of cells (a) under no treatment, (b) under single dose of radiation with 12.5 Gy and (c) under fractional radiation starting at time = 400 h (5 fractions of 2.5 Gy).

To study cell-cycle dynamics when cells are treated with multiple fractions of radiation doses of radiation, we have simulated the radiation therapy of the tumour cells with 2.5 Gy/day for 5 days, up to 12.5 Gy starting at time = 400 h. This is compared against the results of irradiation with a single dose of 12.5 Gy given at time = 400 h and the control cell distribution. The total number of cells and the number of cells that are in G1, G2, and resting phases for (a) the control case, (b) when cells are treated with a single dose of radiation and (c) when cells are treated with fractional doses of radiation, are plotted against time in Figures 2a, 2b and 2c, respectively. The subplots in these Figures show the percentage of hypoxic area with respect to time. It can be seen from Figure 2a that the cells grow mostly in an asynchronous pattern with almost an equal proportion of cells in either G1 or G2 phases, which further tends to reach a plateau as time increases. However, as the tumour size increases, the population of cells in the quiescent phase increases steadily compared with the proliferating population of cells. Although in the present model the cell-cycle delay due to hypoxia and space-limitation contribute to these population dynamics, this effect has previously been related to the basic Gompertzian tumour growth dynamics [25]–[27]. In particular, Gyllenberg and Webb examined the role of cellular quiescence on the pattern of tumour growth and suggested that the basic Gompertzian tumour growth can be explained by a non-linear relationship where the cells in larger tumour masses have an increased probability to enter the quiescent phase [25]. From a clinical perspective, tumours with a large proportion of quiescent cells are much harder to treat than tumours with more of the dividing fraction and most of the time the quiescent cell population is found to be more resistant to most available treatments. Hence, it would be interesting to study these tumour dynamics under various combinations of treatments.

When the cells are irradiated with a single dose of radiation, as discussed in the previous paragraph, the majority of the cells stay in G2 phase of the cell-cycle after the radiation for a short period and then the majority move into G1, stay in that distribution for about 60–70 h (partial synchronisation) before eventually recovering and following the cycling pattern seen in the case of the control cell population (Figure 2b). Note from the subfigure of Figure 2b that when cells are killed, the changing spatial distribution of the tumour mass and the availability of free space reoxygenate the tumour (hypoxia area reduced) as observed experimentally [28], [29], which may further increase the proliferation, but also change radiosensitivity, as hypoxic cells are relatively radioresistant. However, when multiple doses of radiation are given to achieve the same total dose, the synchronisation is observed only during the treatment time, and is lost as soon as the radiation is stopped (Figure 2c). This is consistent with a laboratory experimental result which showed that the distribution of cells is different if the cells are irradiated with either a single large dose or fractionated treatment (giving the same total dose) [30]. Also note that soon after the radiation, with the creation of empty space and a favourable microenvironment, the number of cells in resting phase decreases as the resting cells re-enter the active phase of the cell-cycle (into G1 phase).

#### Hypoxia and radiation

The relative presence or absence of oxygen within the vicinity of a cell greatly influences its cell-cycle status and radiation sensitivity [22], [31]. When oxygenation increases, the biological effect of the ionizing radiation also increases as the presence of oxygen allows the radiation to cause more damage to the tumour cell's DNA [22].

**Figure 3:**
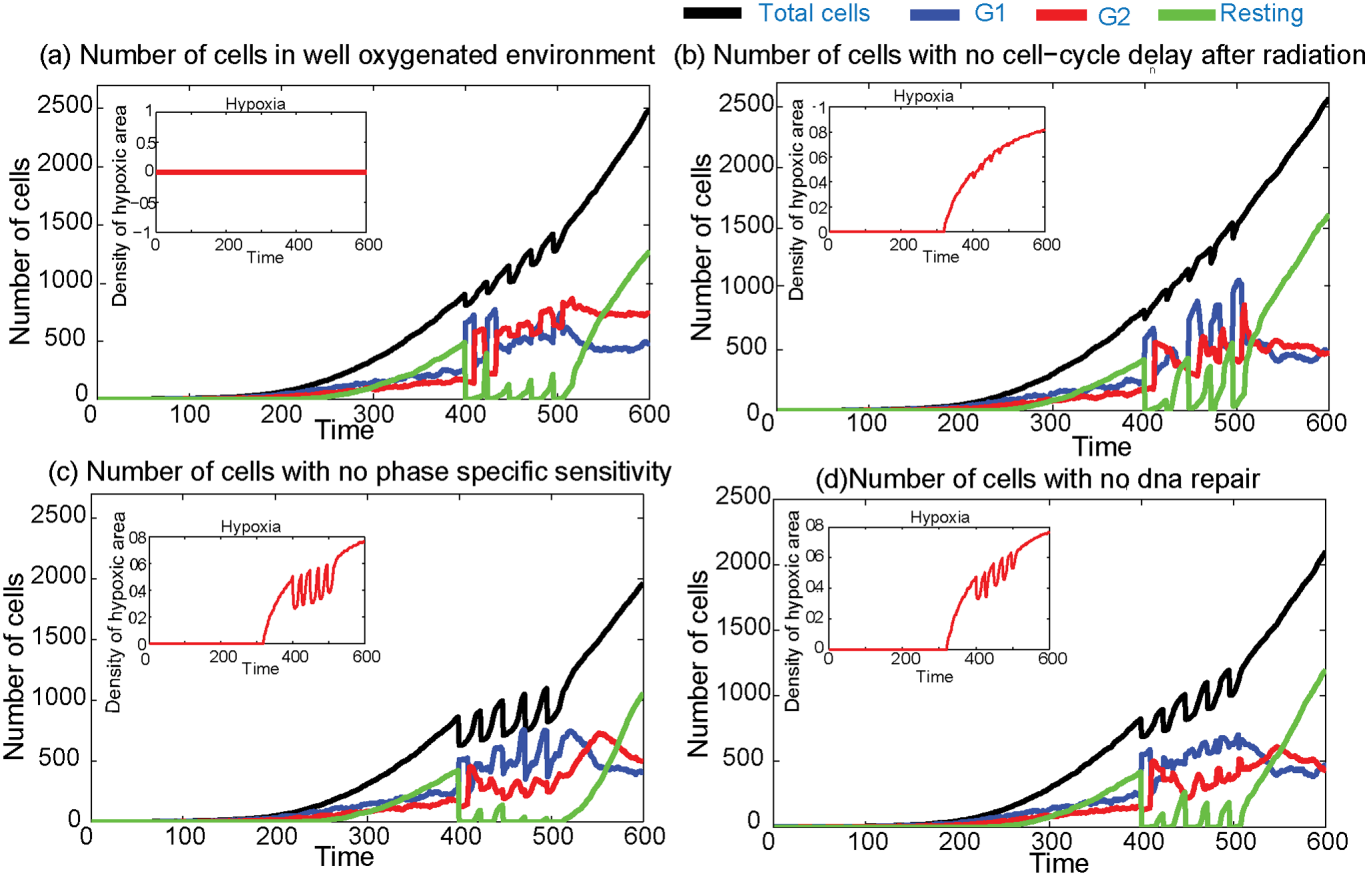
Number of cells under various conditions that influence the radiation damage after the irradiation. (a) Plots for a well oxygenated microenvironment (no hypoxia, OMF = 1 in the equation (12)) (cells killed = 621), (b) plot assuming no cell-cycle delay for repair after the radiation (cells killed = 516), (c) plots assuming there is no cell-cycle phase-specific sensitivity for repair after the radiation (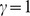 in equation (12)) (cells killed = 1361) and (d)plots when there is no DNA repair after the radiation (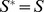 for all doses in equation (13)) (cells killed = 913).

Here, we introduced these effects by using a concept of an oxygen enhancement ratio, which is the ratio of dose to produce a given effect without oxygen to the dose to produce the same effect with oxygen [32]. Furthermore, a well-oxygenated microenvironment can also increase the proportion of proliferating cells. In Figure 3a, we plot the temporal changes in the total number of cells and the cells that are in various phases of the cell-cycle, when cells are irradiated with 2.5 Gy/day for 5 days in a 100% oxygenated microenvironment. Compared to the heterogeneous case in Figure 2c, the presence of oxygen increases the cell-kill, reduces the number of cells in the resting phase and consequently increases the proportion of cells in the proliferative phase with a majority of the cells being in G2 phase at the time of irradiation.

#### Cell-cycle and radiation

As we discussed in the Introduction, an individual cell's radiation sensitivity depends significantly on its cell-cycle status [3], [4]. While the cell-cycle phases determine the radio-responsiveness, radiation itself can modify the cell-cycle dynamics, mainly by delaying the cell-cycle progression to allow repair of DNA damage [4]. In Figures 3b, 3c and 3d, we study various factors that are associated with cell-cycle and radiosensitivity.

In this radiation model, we have assumed that immediately after the radiation, there is a divisional delay of 1–9 h (randomly chosen) if the cells are either in their G1 or G2 phases of the cell-cycle. Figure 3b shows the temporal changes in the number of cells if no cell-cycle delay after the radiation is assumed. Comparing these results with those assuming a repair delay in Figure 2c, it can be seen that the proportion of cells in various phases is slightly different but roughly comparable. This might be due to the fact that the repair time delay is assumed to be less than 10 h, much less than the time interval between two doses of radiation therapy.

In Figure 3c, we have plotted the effects of radiation therapy on the number of cells when no cell-cycle specificity for the radiation sensitivity is assumed. Here, all the cells have the same sensitivity, which is taken to be the maximum (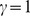 in equation 12). The figure shows that if the radiation sensitivity of the cells is increased there will be a corresponding increase in the cell-kill, which then further enhances the reoxygenation of the tumour (subfigure in Figure 3c).

The number of cells for the case where no DNA repair is assumed is plotted in Figure 3d and shows an increase in the number of cells killed as compared to the normal case (Figure 2c), as expected. In all four cases (Figure 3), when new empty spaces are created by cell death and the microenvironment is well oxygenated, resting cells revert to the normal cell-cycle which further increases the number of cells in the active G1 and G2 phases. Figure 3 also shows that the two important factors that affect the radiation responsiveness of the cells are the cell-cycle phase-specific radiation sensitivity of the individual cells and the activation of the repair mechanisms within the cell.

### The combination of cell-cycle based chemotherapy and radiation therapy

**Figure 4:**
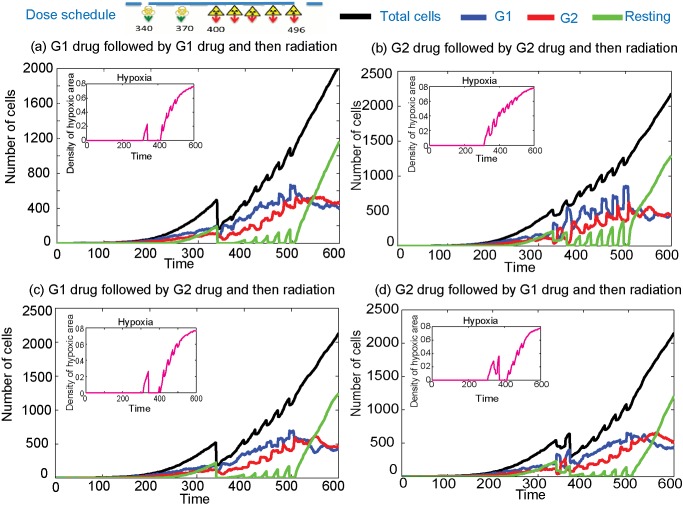
Number of cells when chemotherapy is given before radiation therapy. Two doses of G1 and/or G2 drugs are given at time = 340 h and 370 h, which are followed by 5 fractions of radiation therapy (1 week) with a daily dose of 2.5 Gy starting at time = 400 h. (a) Plots when two G1 phase-specific drugs are given before radiation, (b) plots when two G2 phase-specific drugs are given before radiation, (c) plots when a G1 phase-specific drug followed by a G2 specific drug are given before radiation and (d) plots when a G2 phase-specific drug followed by a G2 specific drug are given before radiation.

Clinically, a kinetically based administration of chemotherapy and radiation therapy is often used to achieve an improved therapeutic effect due to the processes of spatial cooperation, independent additive cell-kill and cellular, molecular and tissue level interaction between modalities [3], [5], [6]. However, most of these interactions are dependent on the type of drugs given and the temporal separation between the drugs and radiation fractions, and hence an appropriate combination of these therapeutic modalities is an essential requirement to achieve maximum survival [33], [34]. Here, we show the analysis of the effects of four hypothetically-scheduled, clinically-used combinations (adjuvant radiation, neo-adjuvant radiation, concurrent radiation and chemo-radiation-chemo) of cell-cycle phase-specific chemotherapy and fractionated radiation therapy. A representative result showing the changing dynamics of cells in various cell-cycle phases for the adjuvant therapy (radiation is given after the chemotherapy) is given in Figure 4 and the figures for the rest of the combinations can be found in the Supplementary Material, in Figure S2, S3 and S4. Moreover, a comparison of the total number of cells for different combination protocols is given in Figure 5.

Figure 4 shows the sequencing of two types of chemotherapeutic drugs followed by radiation therapy. Two doses of cell-cycle phase-specific chemotherapy, specific to either G1 or G2 phases of the cell-cycle, are given at time = 340 h and 370 h, followed by 5 fractions of radiation, given with a daily dose of d = 2.5 Gy, starting at time = 400 h. The plots show that when the radiation therapy is given after the chemotherapy doses, the partial cell synchrony during the radiation is lost and a higher proportion of cells stay in G1, except for the case where two G2 phase-specific drugs (Figure 4b) are combined. In Figure S2, we plot the effects of combination treatments when two doses of the chemotherapy drugs are given after the radiation therapy. The radiation starts at time = 340 h with a similar dose as the previous case and the doses of chemotherapeutic drugs are given at times = 466 h and 496 h. As we have seen in the previous section, the radiation given before two doses of chemotherapy introduces a partial cell-cycle synchrony of cell distribution that remains until the end of the therapy.

The cell-phase distribution for the case when the doses of chemotherapeutic drug are given before and after the radiation, is shown in Figure S3. The figure shows that the administration of a G2-specific drug, which kills fewer number of cells compared to the G1-phase-specific drug, helps to keep the cells in synchrony throughout the treatment time (similar to Figure 4). In the last case, we studied the application of chemotherapy during the radiation schedule where the chemotherapeutic drugs are given at times 370 h and 400 h with the radiation, starting at time = 340 h. The plots in Figure S4 indicate that the increased cell-kill further reduces the cell-cycle synchrony with the number of G1 phase cells being dominant. The total number of cells for all four cases of radiation and chemotherapy sequencing are compared in Figure 5. The plots show that in the absence of additional fractions of radiation and further doses of chemotherapy all the schedules perform in a similar fashion, although some give a better cell-kill. However, the effects of the sequencing are critically dependent on the number of cells in various phases of the cell-cycle, as this determines how sensitive they are to the various therapeutic strategies.

In every combination except concomitant therapy (chemotherapy given during radiation), we kept the total treatment time constant, if not identical, to compare the effects on tumour control. We have also used the same set of parameter values and doses for each phase-specific chemotherapy [15]. It can be seen from Figure 5 that when a G1-phase-specific drug is administrated, the cell-kill is usually higher than a G2-phase-specific drug. This is mainly because, in most of the cases, the percentage of cells that are in G1 phase is higher than those in G2. The two factors that contribute to such a cell population distribution are hypoxia and space limitation, as hypoxic cells take a longer time to complete one full cell-cycle and the lack of space forces the cell to enter a resting phase. The cell will re-enter the active phase of the cell-cycle when conditions become favourable. In other words, resting tumour cells may, under favourable conditions created by the administration of the therapies, play a vital role in cell synchronisation and knowledge of this should inform the design of better combinations of cell-cycle phase-specific chemotherapy and fractionated radiation therapy. Moreover, it can be seen from Figure 5 that although the various combination regimes mostly show varying results immediately after the treatment, in the absence of further treatments, these lead to a similar end point with same number of cells as time increases as observed in most clinical situations. However, these differences in the proportion of cells in various cell-cycle phases immediately after each treatment protocol might be vital in designing further treatment plans for that individual patient.

Additionally, another factor that plays an important role in therapeutic intervention is the spatial distribution of cells in the tumour mass and its blood supply as these determine the nature of the tumour microenvironment. The tumour microenvironment, in particular oxygen distribution, can significantly affect a cell's radiation sensitivity and thereby introduce cell-cycle heterogeneity throughout the tumour. On the other hand, the vascular distribution within and surrounding the tumour mass determines the effectiveness of the spatial distribution and supply of the chemotherapeutic drugs which determine the cell-kill. A representative figure showing the spatial distribution of the cells in various cell-cycle phases at different treatment time points is given in Figure S5.

### Towards a predictive clinical tool: Using the computational model to derive optimal therapeutic regimes

In this section, we illustrate the potential of our multiscale computational model to compare different treatment regimens and test the predictions against what happens in a real tumour model in a prospective manner. In doing so we provide a ranking of each regimen in terms of overall treatment efficacy. This highlights the potential of our model to produce an optimal treatment regimen for a given patient. We have compared two different treatment protocols currently used in oesophageal cancer, namely Herskovic, modified Herskovic (both currently used in clinical practice) and also a third experimental protocol which is currently not used in clinical practice.

Oesophageal cancer is in the “top 10” most common malignancies worldwide, and is the fifth highest in terms of mortality [35]. The treatment of oesophageal cancer used to be primarily surgical or palliative. In the 1980s, clinical trials started that were designed to look at radiation treatment of these tumours and determine whether better results could be obtained by combining radiation therapy with chemotherapy. A seminal trial that commenced in that decade (“Herskovic”) reported two groups of patients who received either radiation therapy alone or radiation therapy with chemotherapy [36]–[38]. Although the trial was not conducted with the same rigour of a modern-day trial (numbers were relatively small and not all the patients were randomized, leading to the possibility of confounding the results), the authors reported that none of the radiotherapy alone group but a significant minority of the combined modality group was alive several years after treatment. The method of giving “chemo-radiotherapy” for non-operable cases was then adopted and a dose of radiation and chemotherapy using Cisplatin and 5-FU was used as most patients could tolerate this combination without severe or life-threatening complications. A later modification, not supported by any trial evidence, was to change the extra chemotherapy given in this regimen from adjuvant (after the chemo-radiotherapy, to neo-adjuvant (before the radiotherapy) as this was better tolerated [38]. This “modified-Herskovic” regimen was then used for the best part of a decade or more, before further clinical studies were done to evaluate new chemotherapy agents and different radiotherapy planning techniques [38]. One of these, the SCOPE-1 study, a two arm, open, randomised multicentre Phase II/III trial, is designed to investigate the effect of the drug cetuximab on chemoradiotherapy [39]. This study has recently stopped recruiting and involves hundreds of patients.

For the Herskovic treatment protocol, chemotherapy treatment is given on weeks 1, 5, 8 and 11 of the treatment period with Cisplatin 80 mg/m2 on day 1 (D1) as a single dose, and 5FU, 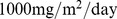 from day 1 to 4 (D1-4) as a continuous infusion. Radiation therapy is given from weeks 1 to 5, in 25 fractions of 2 Gy. Similarly, for the modified Herskovic regimen, chemotherapy is given on weeks 1, 3, 6 and 10 and radiation therapy is given from week 6 to 10 in 25 fractions of 2 Gy dose. Finally, for the experimental protocol, which has been designed for this comparison study, chemotherapy is given on weeks 1, 3, 6 and 9 and radiation is given from weeks 12 to 15 in 20 fractions of 2.5 Gy dose. Note that, in all these three treatment protocols the total amount of chemotherapeutic drug and the radiation dosage is kept the same (although their biological effect are different).

**Figure 6:**
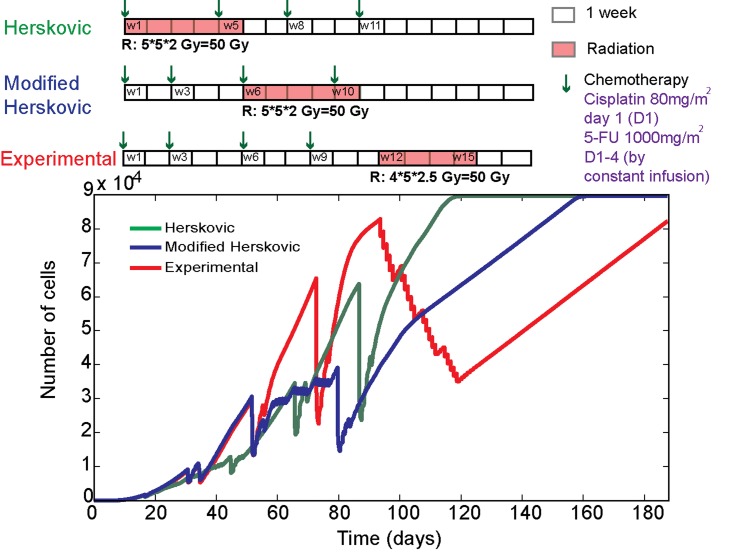
Plots showing the computational simulation results of our multiscale model comparing the outcomes of three different treatment regimens over a period of 17 weeks for patients with oesophageal cancer. Herskovic (green line), Modified Herskovic (blue line) and Experimental (red line) [see text for details of each regimen]. The results show that the Modified Herskovic treatment protocol gives a better final outcome than that of the Herskovic treatment plan. However, the model also predicts that the newly proposed experimental treatment protocol is better than both the Herskovic and Modified Herskovic.

We simulated treatment with each of the three regimens using our multiscale model over a period of 17 weeks. The simulations were carried out using precisely the same set of parameter values for all three treatment regimens and the results showing the total cell kill over time are given in Figure 6. First of all we note, as can be seen by comparing the green and blue curves, that our computational simulation results show that the modified Herskovic regimen gives a better final outcome than that of the Herskovic treatment plan as suspected clinically [38]. This gives a degree of confidence in our multiscale model, since this is effectively a “blind test” between the two different regimens. However, interestingly, as seen by comparing the red curve with the green and blue curves, our multiscale model predicts that the new experimental regimen is more effective than either the Herskovic or Modified Herskovic regimen. Although more clinical and experimental studies would be required to confirm these predications, these results highlight the predictive power of our model and its ability to distinguish between a number of different regimens and rank them in terms of overall efficacy or indeed even to predict an optimal treatment strategy.

### Conclusions

One of the crucial steps towards the successful delivery of anti-cancer treatment is the optimal scheduling and sequencing of different therapeutic modalities, in particular radiotherapy and chemotherapy. The roles of cell-cycle phases as well as tissue hypoxia are believed to be critical in determining the radiation sensitivity of cells and the action of several chemotherapeutic drugs [3]. Changes in the treatment of cancer are currently driven largely by the products emerging from the pharmaceutical industry and although some thought and time is devoted to understanding how best to schedule, combine, and deliver the anti-cancer treatments that are currently available in order to increase their effectiveness, it would be helpful to be able to make rapid rationale treatment choices when designing new treatments based on currently available knowledge rather than take several years to test two treatment regimens in the clinical setting, as currently happens with clinical phase III studies. We believe *in silico* experiments may help in this regard.

In this paper, we have presented a hybrid multiscale cellular automaton model to study the effects of radiotherapy, alone and in combination with cell-cycle specific chemotherapeutic drugs, in controlling the growth of a solid tumour. We have also incorporated the heterogeneities in cell-cycle dynamics and oxygen distribution into our hybrid cellular automaton model as they play an important role in therapeutic effectiveness. The effect of radiation therapy is studied using a modified linear quadratic model that incorporates some of the important factors responsible for radiation sensitivity such as cell-cycle phase-specific radiation sensitivity, improved survival due to DNA repair, and hypoxia. The simulation results from the model showed very good agreement with previous biological experimental results in predicting the cell-cycle dynamics after the irradiation of the tumour cell mass with a single dose of the radiation. The results predicted an increase in the number of G2 phase cells and a possible scenario of partial synchronisation of the cell-cycle, while the control cell population remained in a more or less G1 phase cells dominated proportion. When the cells are irradiated with fractionated radiation, the results showed that the cell-kill enhances the reoxygenation of the tumour mass but also allows the re-entry of resting cells into the active cell-cycle.

The study of various factors affecting radiation sensitivity indicated that cell-cycle phase-specific sensitivity and survival due to DNA repair mechanisms could play a vital role in improving radiation cell-kill. Using the present model we have also analysed various possible combinations of cell-cycle phase-specific chemotherapeutic drugs and fractionated radiotherapy. The results show that the sequencing and the type of the chemotherapeutic drugs can significantly affect the cell-cycle and oxygen heterogeneities of the tumour mass which will further affect the effectiveness of the entire therapeutic strategy when they are given in several doses and/or fractions and are consistent with various experimental results [33], [34]. Overall, the results from the model show its potential usefulness in studying and understanding a kinetic administration of cell-cycle phase-specific chemotherapeutic drugs in combination with radiation therapy. The results from the current and previous studies [15] also confirmed the importance of temporally changing spatial dynamics to improve therapeutic strategies. In future studies, we would like adapt the current model to address the interactions between tumour cells and normal cells and to study how their combined spatial dynamics affect their therapeutic responses.

Furthermore, our general computational model can be easily adapted to reflect the behaviour in real clinical scenarios by appropriate validation and comparison with experimental and clinical data. One such comparison on the treatment protocols used in the treatment of oesophageal cancer indicated a therapeutic benefit of the modified Herskovic treatment protocol over the previously used Herskovic protocol. Moreover, the simulations indicated that a suggested new experimental treatment protocol might be an even better strategy than currently used treatment options, but clearly more detailed studies are necessary to validate this prediction.

As discussed earlier, the evolution of clinical treatment is slow and takes place over many decades, for the reasons that clinicians must be cautious when introducing new treatments in case of poor efficacy or excess and unexpected toxicity or intracellular and extracellular heterogeneities, and that once a preferred treatment route has been started on, it is generally modified in an incremental fashion. It is very unusual for clinicians to start a completely new way of treating tumours without evidence. The computaional simulation results of our multiscale mathematical model indicates a way for doctors to test the efficacy of new treatment strategies, to allow them to plan more adventurous treatments *in silico*, prior to beginning actual testing and long and costly clinical trials. This departure may help relieve some of the stagnation in treatment strategy for tumours that have a poor prognosis, and allow medicine to move forward to more innovative treatments that can be evaluated for potential efficacy, prior to clinical testing.

## Methods

**Figure 7:**
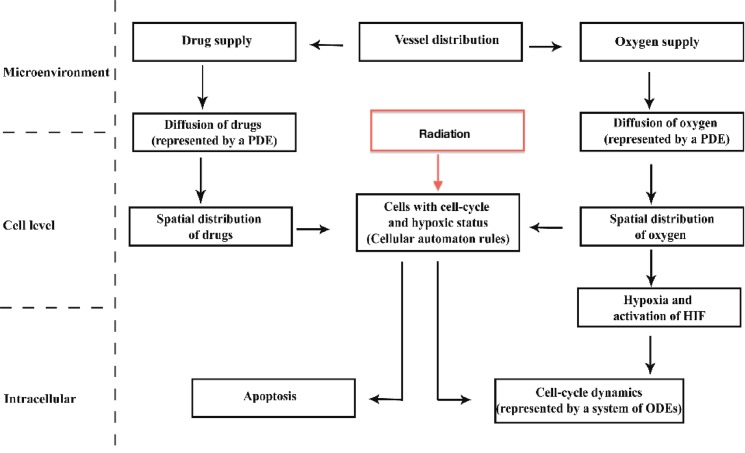
Schematic diagram of the model showing the appropriate scales involved.

The computational experiments are performed on a two dimensional spatial computational grid using a previously developed hybrid multiscale cellular automaton model, incorporating the effects of radiation in combination with the chemotherapy [15]. To study the spatio-temporal growth of the cancer cells and their response to the radiotherapy and chemotherapy, the model consists of four major components that associates each automaton cell. These are: (1) cells - the automaton element is occupied either by a cancer cell or it remains empty. If the automaton (grid) cell is occupied by a cancer cell, the automaton rules that control the evolution of this cancer cell are mainly based on a system of ordinary differential equations that control the cell-cycle dynamics; (2) the local oxygen (hence hypoxia inducible factor1−α (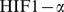)) concentration, whose evolution is modelled by a system of partial differential equation; (3) randomly distributed blood vessels from where the oxygen is supplied within the domain and (4) chemotherapeutic drug concentrations, modelled by a system of partial differential equations. A schematic overview of the model with the scales involved is given in Figure 7. A brief summary of each of these components are given in the following subsections and more details of this hybrid multiscale cellular automaton model (excluding the radiation model) and the parameter values can be found in Powathil et al. [15]

### Cellular and intracellular level

The computational model is simulated on a spatial grid of size 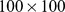 grid points and each automaton element whether it is empty or occupied, has a physical size of 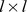, where 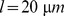, simulating a cancer tissue of 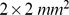 area. If the element is occupied by a cancer cell, the evolution of this cancer cell is based on the decisions made by the cell-cycle mechanism within the cell. To model the cell-cycle dynamics within each cell, we use a very basic model originally developed by Tyson and Novak [40], [41] that includes only the interactions which are considered to be essential for cell-cycle regulation and control, as given below.
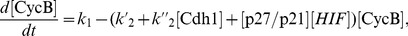
(1)

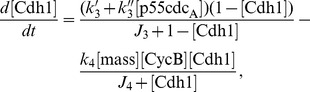
(2)

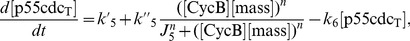
(3)

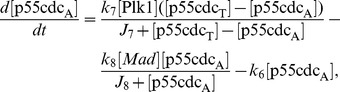
(4)

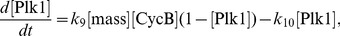
(5)

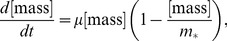
(6)where 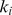 are the rate constants and the values are chosen in proportional to those in Tyson and Novak [40], [41] as given in Powathil et al. [15].

In addition to the cancer cells and the empty spaces, the spatial domain also consists of a random distribution of blood vessel cross sections with density 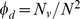, where 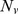 is the number of vessel cross sections (Figure S6). This can be justified if we assume that the blood vessels are perpendicular to the cross section of interest and there are no branching points through the plane of interest [42], [43]. Moreover, the tumour vessel network is irregular, chaotic and abnormal as compared to that of normal vascular network [44], [45].

## Microenvironment and tumour growth

The effects of microenvironment in the progression of tumour growth is included in the model by incorporating the oxygen dynamics, which are modelled using the following partial differential equation.
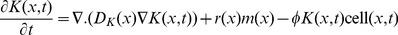
(7)where 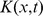 denotes the oxygen concentration at position 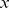 at time 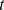, 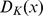 is the diffusion coefficient and 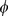 is the rate of oxygen consumption by a cell at position 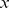 at time 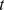 (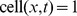 if position 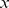 is occupied by a cancer cell at time 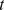 and zero otherwise). Here, 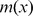 denotes the vessel cross section at position 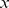 (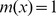 for the presence of blood vessel at position 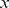, and zero otherwise); thus the term 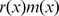 describes the production of oxygen at rate 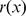. Here, the diffusion coefficient and the supply rate of the oxygen vary depending the location of the of the cancer cells and blood vessels as explained in [15]. This equation is solved using a no-flux boundary conditions and an initial condition [32].

The changes in the oxygen concentration, especially hypoxia may affect various intra and intercellular process of the cells that constitute the tumour mass. In the present model, the effects of hypoxia are included through the activation and inactivation of 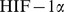 which further results in changes in intracellular cell-cycle dynamics. When oxygen concentration at a specific position 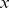 falls below 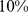 (hypoxic cell), 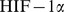 is assumed to become active from an inactive phase, which further delays the cell-cycle dynamics (cf. Equation (1)). Here, we assume that all the cancer cells consume oxygen and do not exhibit Warburg effect and also ignore other transcriptional responses of HIF1 such as glycolysis and angiogenesis.

### Chemotherapy

Chemotherapy is a commonly used treatment for cancer. Chemotherapeutic drugs act on rapidly proliferating cells, such as cancer cells, by interfering with the cell-cycle and other cell-cycle specific targets. Hence, it might be more effective to use a combination of chemotherapeutic drugs that targets the cells in different phases of the cell-cycle. The distribution of chemotherapeutic drug type 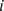, 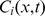 can be modelled by a similar equation as that of oxygen distribution (Eq. 7), given by:
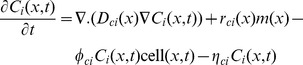
(8)where 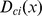 is the diffusion coefficient of the drug type 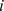, 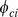 is the rate by which the drug is take in by a cell (assumed to be zero as it is very negligible when compared to oxygen uptake), 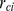 is the drug supply rate by the pre-existing vascular network and 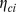 is the drug decay rate [32]. Here, as similar to the oxygen distribution, the diffusion 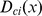 and the supply rate 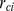 of the drugs are spatially varied depending on the location in the computational domain. The details can be found in [15].

### Radiation response

Radiation therapy is often used in combination with chemotherapy with the intention of increased therapeutic gain for patients with cancer. The survival probability of the cells after they are irradiated are traditionally calculated using linear quadratic (LQ) model [46], given by
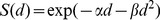
(9)where 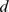 is the radiation dose and 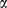 and 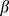 are sensitivity parameters, taken to be 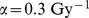 and 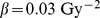 [32]. It has been observed that the radiation sensitivity varies with the cell's oxygenation status [47], [48] and the effect of changing tissue oxygen levels on the radiation sensitivity can be incorporated into the LQ model (Eq. 9) by using the concepts of an “oxygen enhancement ratio” or “oxygen modification factor” [32], defined as
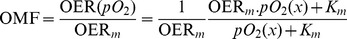
(10)where 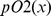 is the oxygen concentration at position 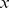, OER is the ratio of the radiation doses needed for the same cell kill under anoxic and oxic conditions, 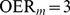 is the maximum ratio and 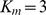 mm Hg is the pO2 at half the increase from 1 to 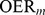 [32], [49]. Hence, the modified LQ model for the survival probability, incorporating the effects of oxygen distributions can be written as:
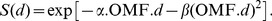
(11)


The relative radiosensitivity of an individual cell is also partially determined by the cell's cell-cycle phase and studies show that the cells are more sensitive when in the S-G2-M phase as compared with the G1 phase [3]. We have incorporated this varying sensitivity due to the changes in cell-cycle phase by an additional term 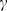 in the equation for survival probability (Eq. 11) [20], which gives:
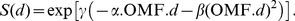
(12)The parameter 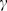 varies from 0 to 1, depending on the individual cell's position at the time of the irradiation. Here, we assumed that the cells in S-G2-M phase has maximum sensitivity with 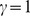 while the cells in G1 phase and the resting phase has relative sensitivities of 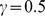 and 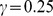, respectively.

In the current model, although we are not considering the individual cell repair, the studies suggest that 98% of damage caused by the radiation is likely to be repaired within few hours of radiation, if they are treated with low dose radiation (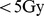) [50], [51]. If the radiation dosage is higher, this repair mechanism may not be sufficient to repair all the DNA damage. Considering these aspects, Endering et al. [16], introduced some correctional terms into the LQ model to accommodate these effects due to the cellular repair during low dose radiation treatment. Using these modifications (allowing for less repair) into the above LQ model (Eq. 11) with the effects of hypoxia, we obtain the survival probability of the cell as:
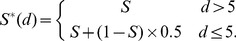
(13)This survival probability is then used to calculate the survival chances of each cell when they are irradiated with the radiation rays. To study this survival chance of an individual cell, a random number is drawn for each cell at every time when they are irradiated and compared against the calculated survival probability. The irradiated cell survives if the random number is smaller than the survival probability and die otherwise.

Here, we also consider the effects of radiation on cell-cycle as irradiation results in a divisional delay, and, in particular, G2 phase delay/arrest in many cell lines [3], [4]. Experimental results show that irradiated cells in G2 phase may take up to 9 hours longer to complete the cell-cycle due to the activation of several intracellular repair mechanisms induced by the radiation [4]. Radiation damage can also induce a cell-cycle delay in G1 phase, mainly through the activation of p53 and p21 pathways [3]. In the present model, we include this effect of irradiation induced delay by forcing the cells to stay in the same phase for an extra time duration of up to 9 hours [4]. This divisional delay might be an important factor to consider while studying the optimal sequencing of radiation therapy with cell-cycle phase-specific chemotherapy.

### Computational methods

**Figure 8:**
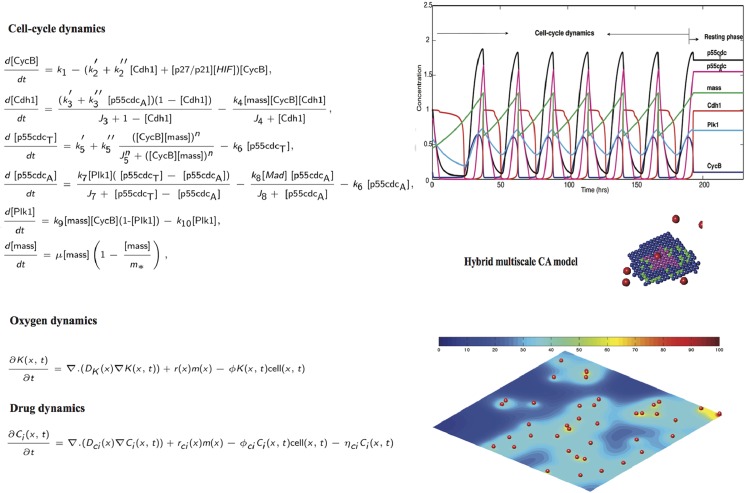
Figure showing various processes involved in the simulation. Plot of the concentration profiles of the various intracellular proteins and the cell-mass over a period of 200 hours for one automaton cell in the model. This is obtained by solving the system of equations, Equations 1 to 6 with the relevant parameter values from Powathil et al. [15] and the plot below shows a representative realisation of the spatial distribution of oxygen or drugs, obtained by solving the corresponding equations.

The hybrid multiscale cellular automaton model is simulated using the rules and the parameters that are described in Powathil et al. [15]. Here, the position of the new daughter cells are determined by Moore and Von Neumann neighbourhoods alternatively to avoid the associated cell distribution patterns specific to each method. An overview of the equations and their simulation results as adapted from Powathil et al. [15] is given in the Figure 8. We have incorporated the effects of radiation into the model using the equations (12) and (13). The survival status of an individual cell is then determined using the calculated survival probability by comparing them against a random probability.

## Supporting Information

Figure S1 **Percentage of cells in G1 and G2 states with and without radiation.** (a) experimental results from [23] and (b) simulation results (percentage of proliferating cells).
(TIF)

Figure S2 **Number of cells when chemotherapy is given after radiation therapy.** Two doses of G1 and/or G2 drugs are given at time = 466 h and 496 h, after 5 fractions of radiation therapy (1 week) with a daily dose of 2.5 Gy starting at time = 340 h. (a) Plots when two G1 phase-specific drugs are given after radiation, (b) plots when two G2 phase-specific drugs are given after radiation, (c) plots when a G1 phase-specific drug followed by a G2 specific drug are given after radiation and (d) plots when a G2 phase-specific drug followed by a G2 specific drug are given after radiation.
(TIF)

Figure S3 **Number of cells when a chemotherapy (one dose) is given before and after radiation therapy.** Two doses of G1 and/or G2 drugs are given at time = 340 h and 496 h and 5 fractions of radiation therapy (1 week) with a daily dose of 2.5 Gy are given in between the chemotherapy doses, starting at time = 370 h. (a) Plots when two G1 phase-specific drugs are given, each before and after radiation, (b) plots when two G2 phase-specific drugs are given, each before and after radiation, (c) plots when a G1 phase-specific drug is given before the radiation followed by a G2 specific drug and (d) plots when a G2 phase-specific drug is given before radiation followed by a G2 specific drug.
(TIF)

Figure S4 **Number of cells when chemotherapy is given during radiation therapy.** Two doses of G1 and/or G2 drugs are given at time = 370 h and 400 h, during the 5 fractions of radiation therapy (1 week) with a daily dose of 2.5 Gy starting at time = 340 h. (a) Plots when two G1 phase-specific drugs are given during radiation, (b) plots when two G2 phase-specific drugs are given during radiation, (c) plots when a G1 phase-specific drug followed by a G2 specific drug are given during radiation and (d) plots when a G2 phase-specific drug followed by a G2 specific drug are given during radiation.
(TIF)

Figure S5 **The spatial distribution of cells within a growing tumour before, during and after the combination therapy.** Plots showing the spatial distribution of cells within a growing tumour at (a) time = 340 h, (b) time = 345 h, (c) time = 370 h, (d) time = 420 h, (e) time = 470 h, (f) time = 496 h, (g) time = 500 h and (h) time = 600 h when tumour is treated with two G1 phase-specific drugs are given, each before and after radiation therapy (5 fractions of 2.5 Gy). The colour represents different cell-cycle status of the individual cells, which are G1 (blue), S-G2-M (green), resting (magenta), hypoxic cells in G1 (rose), hypoxic cells in S-G2-M (yellow) and hypoxic cells in resting (silver).
(TIF)

Figure S6 **Plot showing the concentration profile of oxygen supplied from the vasculature.** The red coloured spheres represent the blood vessel cross sections and the colour map shows the percentages of oxygen concentration.
(TIF)
