# Supplementary figures and images for: Towards Predicting the Response of a Solid Tumour to Chemotherapy and Radiotherapy Treatments: Clinical Insights from a Computational Model
Source: PLoS Comput Biol. 2013 Jul 11;9(7):e1003120. doi: 10.1371/journal.pcbi.1003120 (PMC3708873; doi:10.1371/journal.pcbi.1003120)

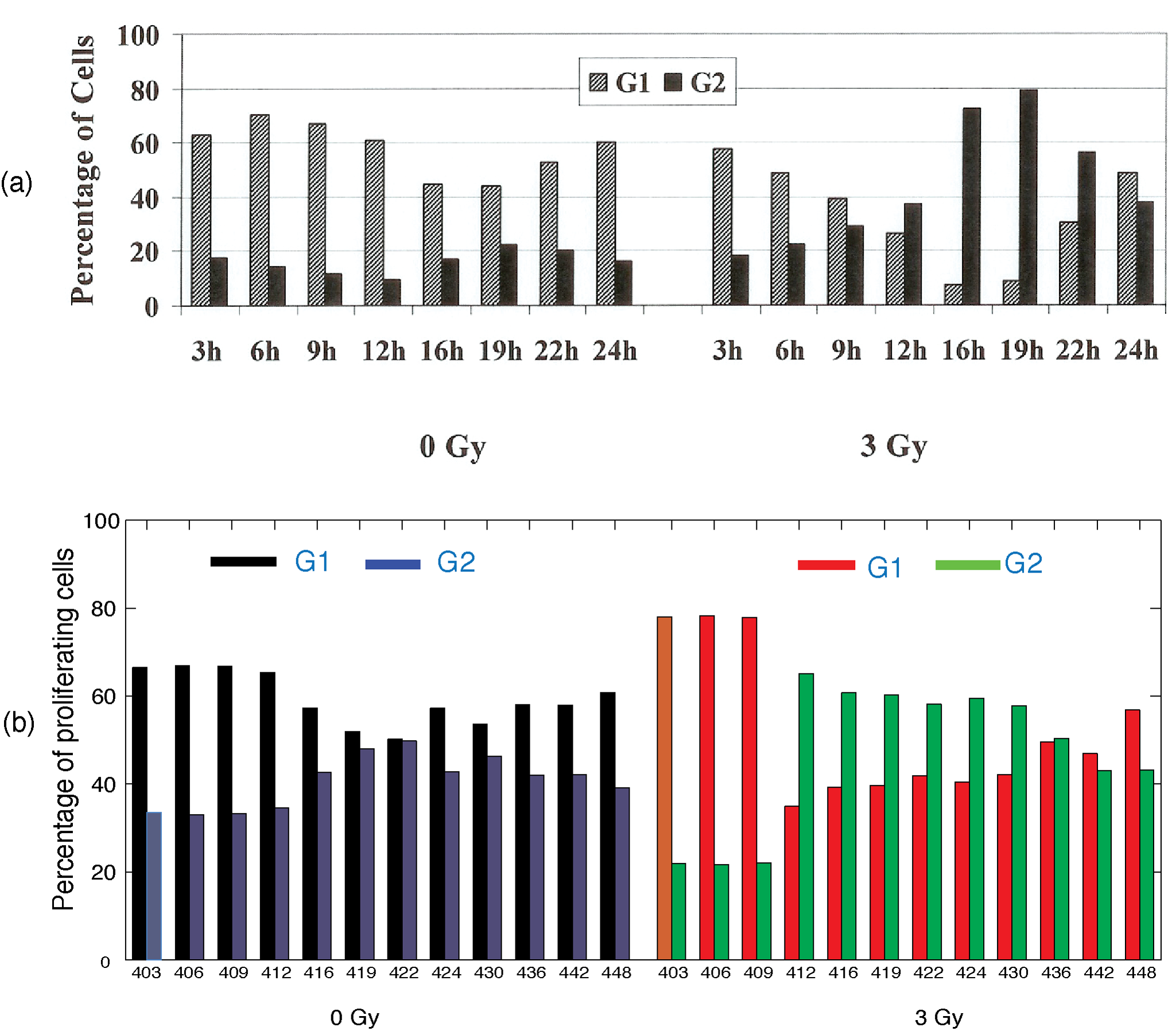

Supplement: Figure S1 — Percentage of cells in G1 and G2 states with and without radiation. (a) experimental results from [23] and (b) simulation results (percentage of proliferating cells). (TIF) [file pcbi.1003120.s001.tif]

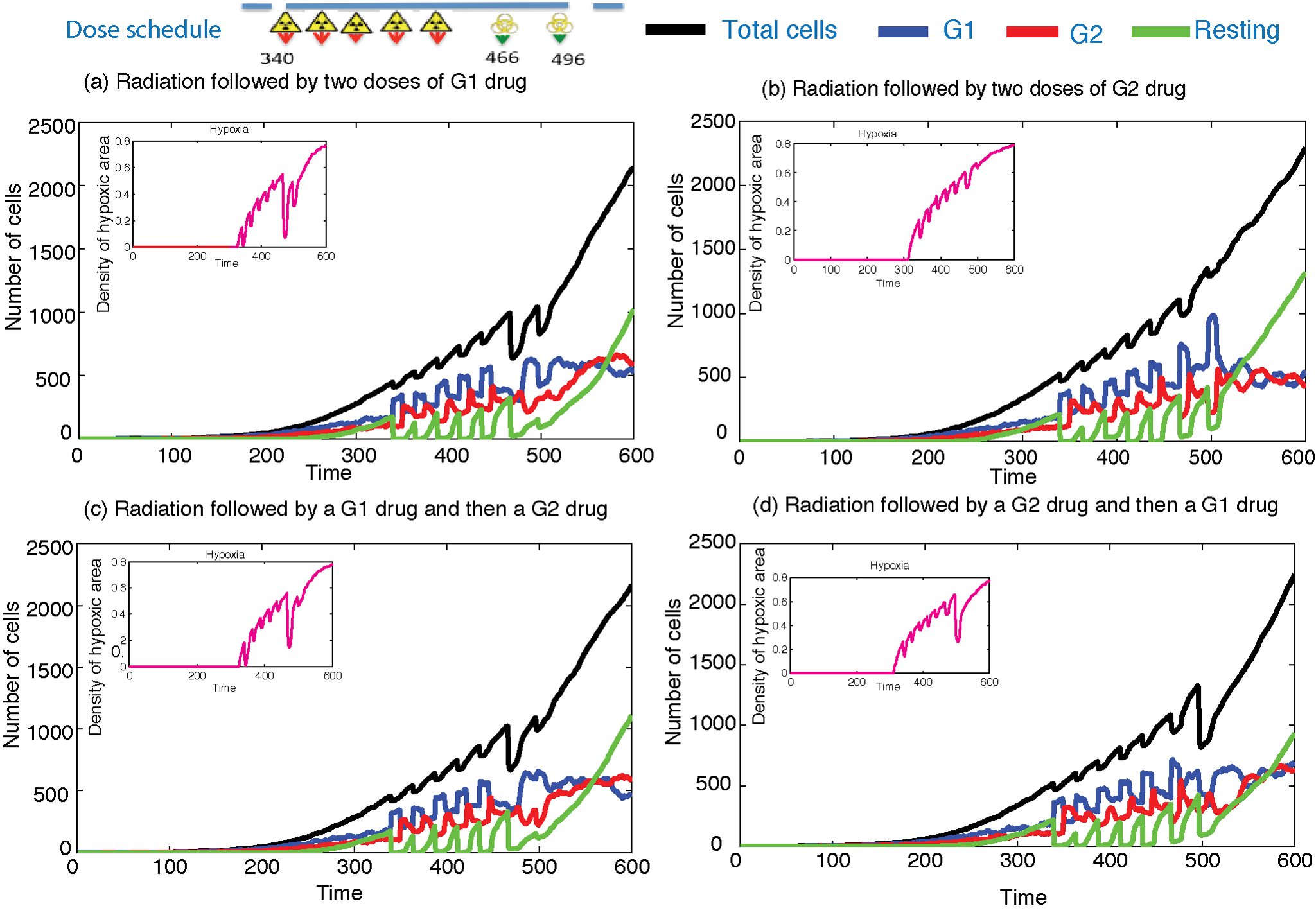

Supplement: Figure S2 — Number of cells when chemotherapy is given after radiation therapy. Two doses of G1 and/or G2 drugs are given at time = 466 h and 496 h, after 5 fractions of radiation therapy (1 week) with a daily dose of 2.5 Gy starting at time = 340 h. (a) Plots when two G1 phase-specific drugs are given after radiation, (b) plots when two G2 phase-specific drugs are given after radiation, (c) plots when a G1 phase-specific drug followed by a G2 specific drug are given after radiation and (d) plots when a G2 phase-specific drug followed by a G2 specific drug are given after radiation. (TIF) [file pcbi.1003120.s002.tif]

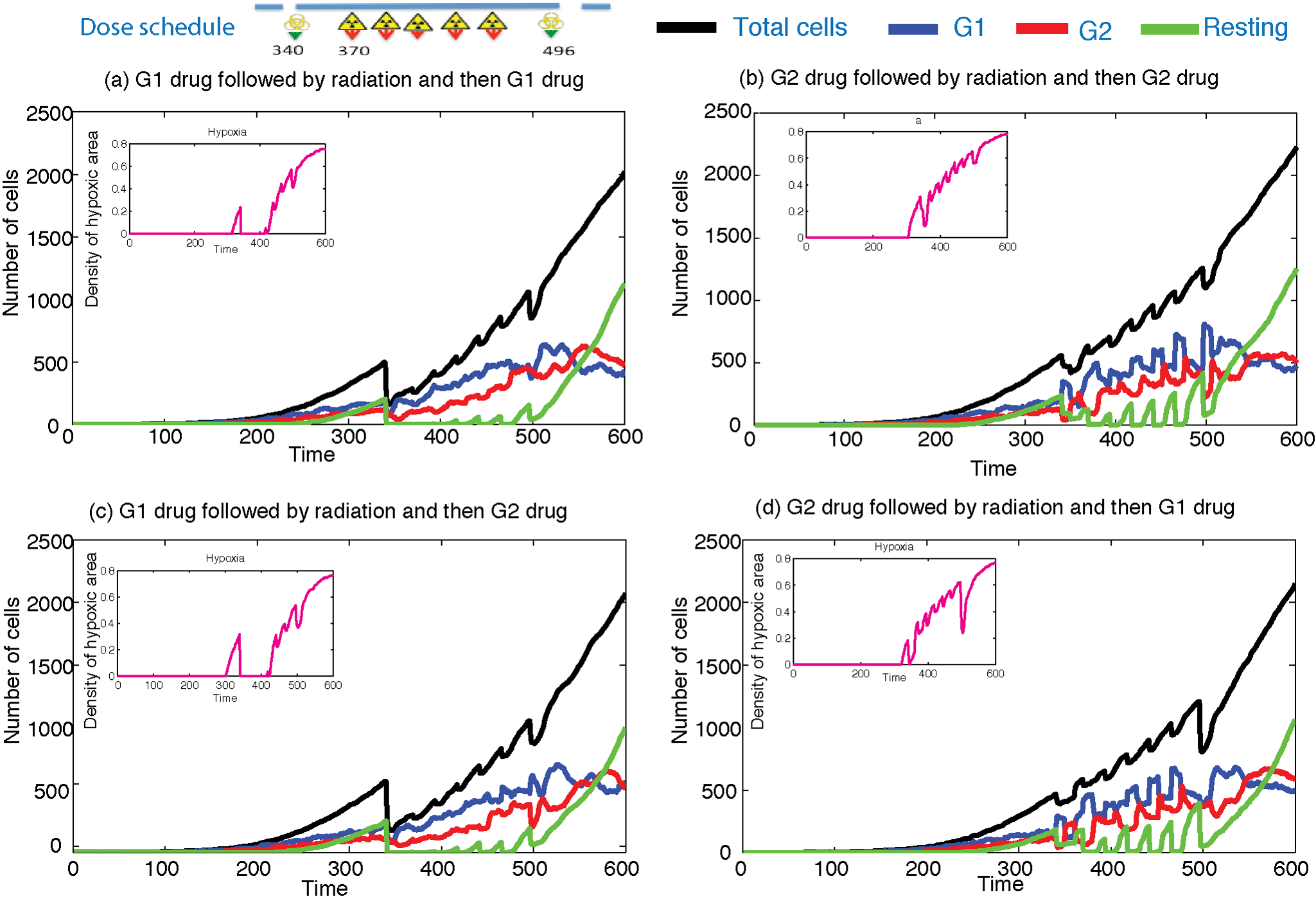

Supplement: Figure S3 — Number of cells when a chemotherapy (one dose) is given before and after radiation therapy. Two doses of G1 and/or G2 drugs are given at time = 340 h and 496 h and 5 fractions of radiation therapy (1 week) with a daily dose of 2.5 Gy are given in between the chemotherapy doses, starting at time = 370 h. (a) Plots when two G1 phase-specific drugs are given, each before and after radiation, (b) plots when two G2 phase-specific drugs are given, each before and after radiation, (c) plots when a G1 phase-specific drug is given before the radiation followed by a G2 specific drug and (d) plots when a G2 phase-specific drug is given before radiation followed by a G2 specific drug. (TIF) [file pcbi.1003120.s003.tif]

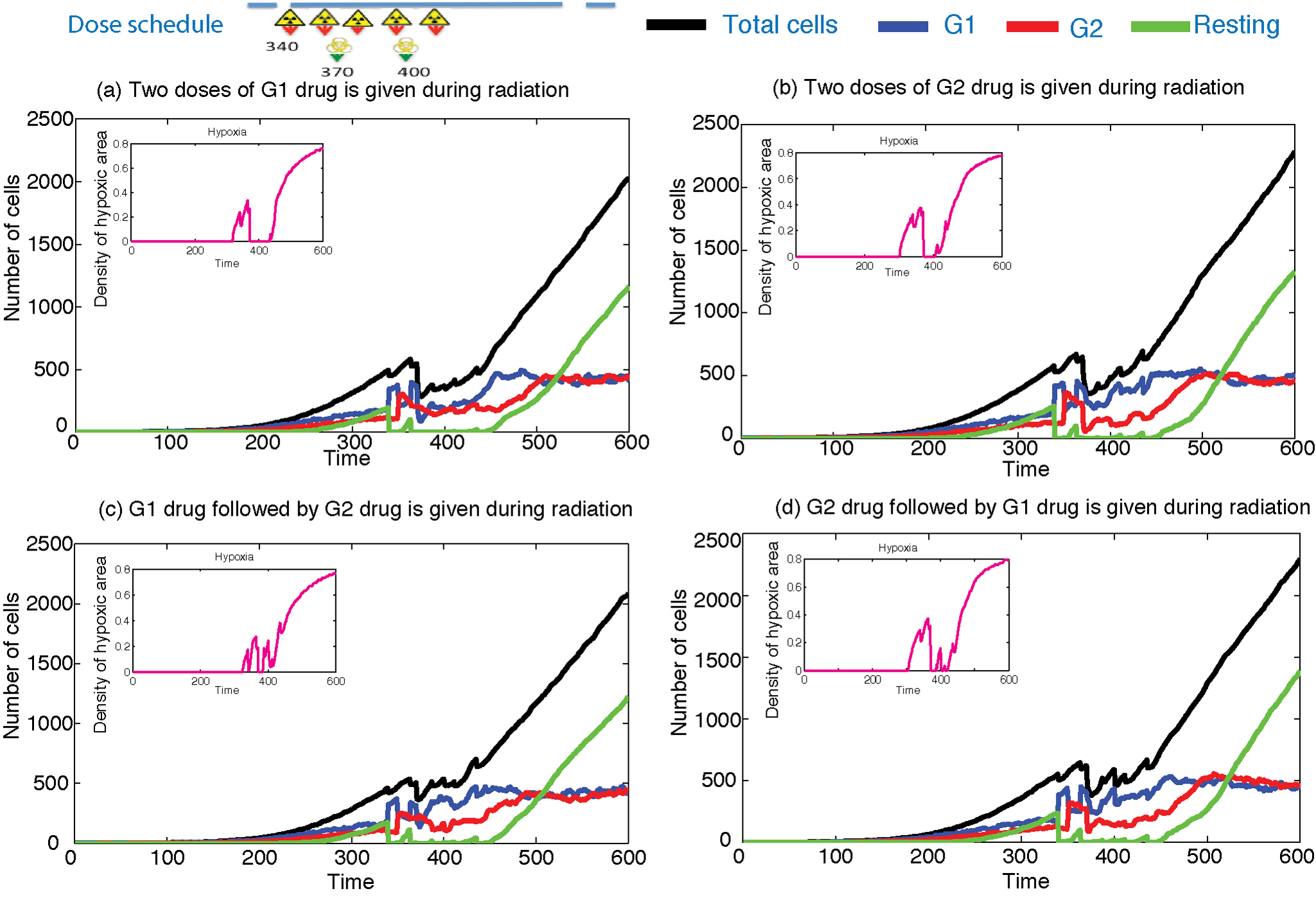

Supplement: Figure S4 — Number of cells when chemotherapy is given during radiation therapy. Two doses of G1 and/or G2 drugs are given at time = 370 h and 400 h, during the 5 fractions of radiation therapy (1 week) with a daily dose of 2.5 Gy starting at time = 340 h. (a) Plots when two G1 phase-specific drugs are given during radiation, (b) plots when two G2 phase-specific drugs are given during radiation, (c) plots when a G1 phase-specific drug followed by a G2 specific drug are given during radiation and (d) plots when a G2 phase-specific drug followed by a G2 specific drug are given during radiation. (TIF) [file pcbi.1003120.s004.tif]

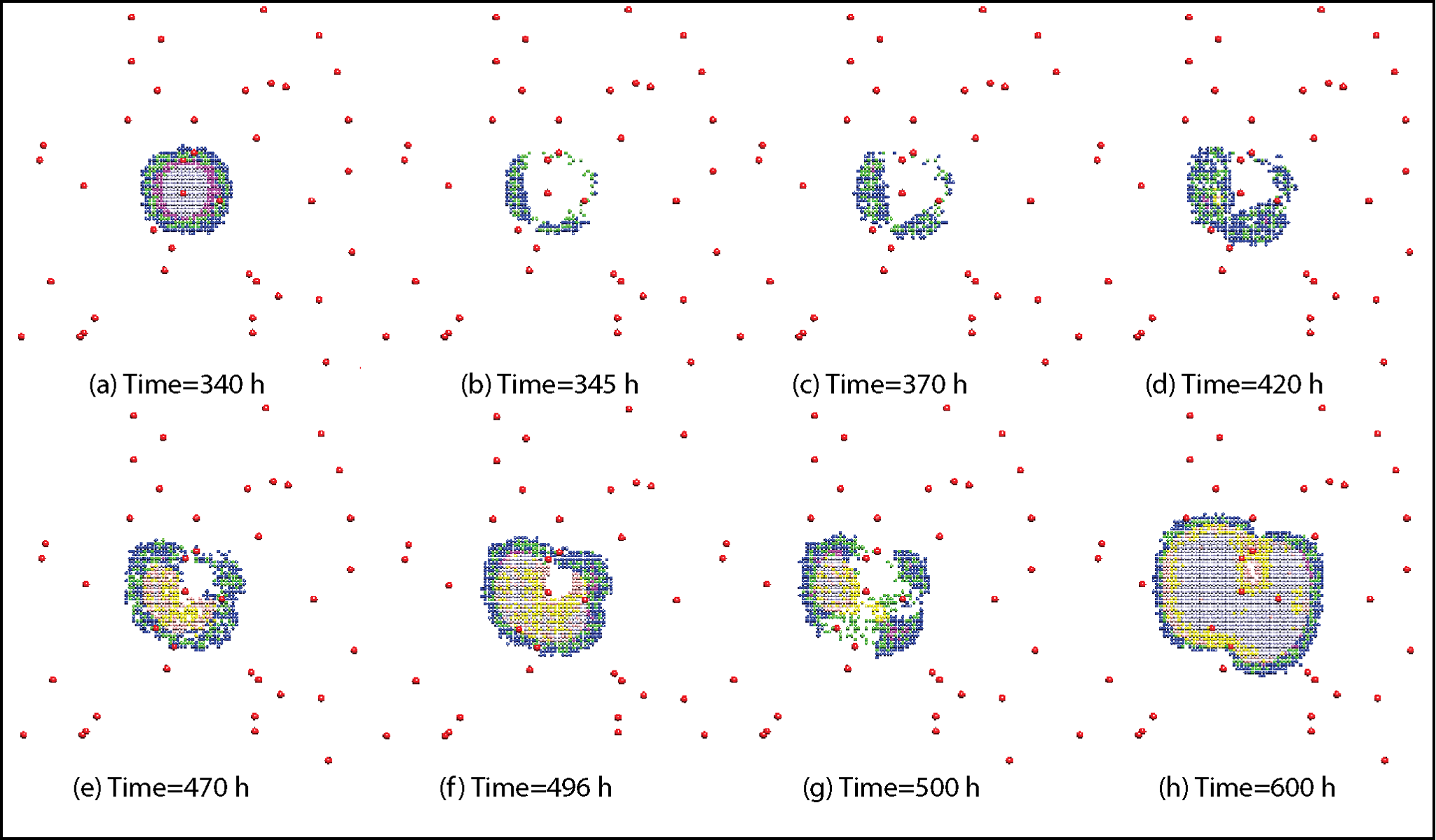

Supplement: Figure S5 — The spatial distribution of cells within a growing tumour before, during and after the combination therapy. Plots showing the spatial distribution of cells within a growing tumour at (a) time = 340 h, (b) time = 345 h, (c) time = 370 h, (d) time = 420 h, (e) time = 470 h, (f) time = 496 h, (g) time = 500 h and (h) time = 600 h when tumour is treated with two G1 phase-specific drugs are given, each before and after radiation therapy (5 fractions of 2.5 Gy). The colour represents different cell-cycle status of the individual cells, which are G1 (blue), S-G2-M (green), resting (magenta), hypoxic cells in G1 (rose), hypoxic cells in S-G2-M (yellow) and hypoxic cells in resting (silver). (TIF) [file pcbi.1003120.s005.tif]

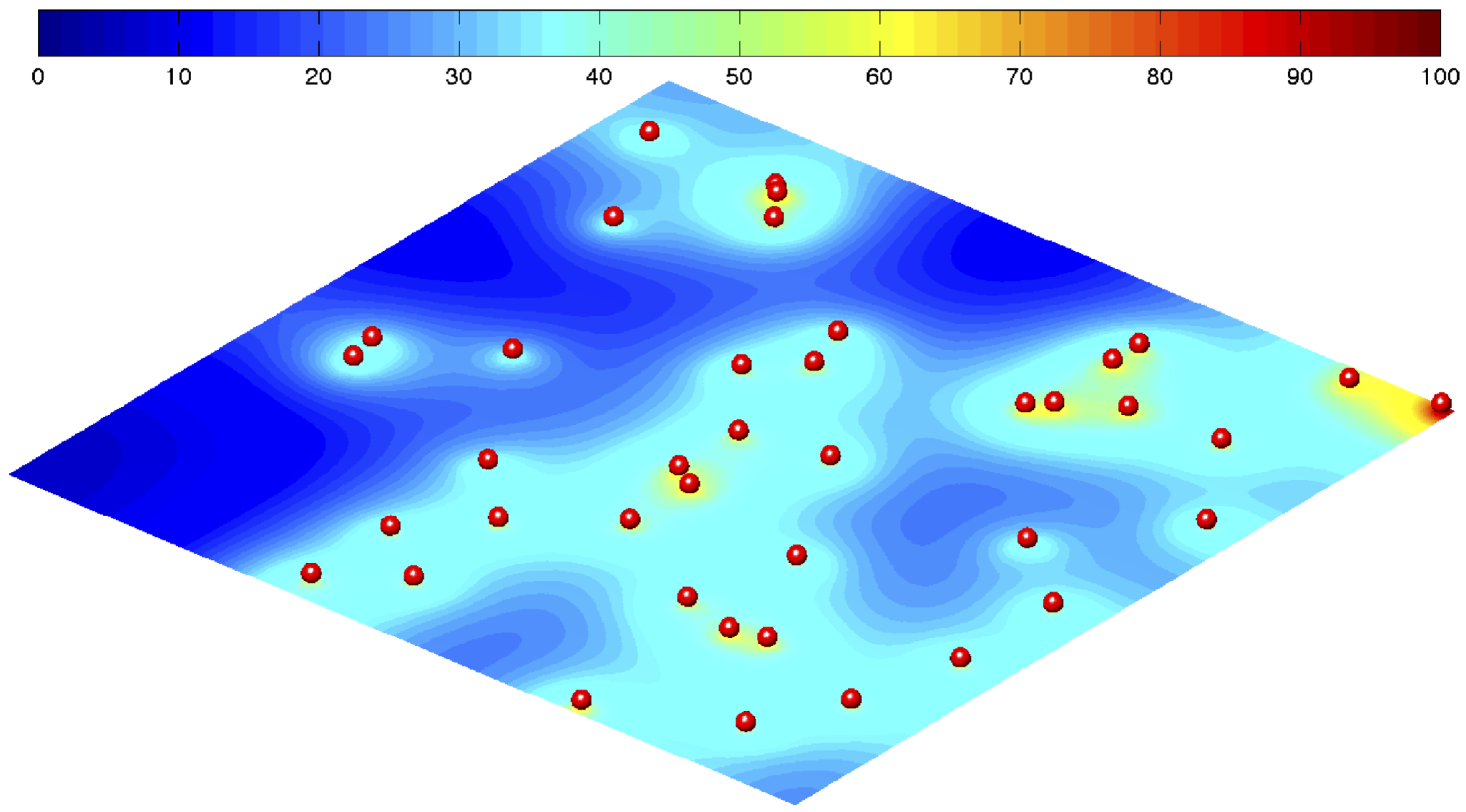

Supplement: Figure S6 — Plot showing the concentration profile of oxygen supplied from the vasculature. The red coloured spheres represent the blood vessel cross sections and the colour map shows the percentages of oxygen concentration. (TIF) [file pcbi.1003120.s006.tif]
